# Prefrontal Cortex Based Sex Differences in Tinnitus Perception: Same Tinnitus Intensity, Same Tinnitus Distress, Different Mood

**DOI:** 10.1371/journal.pone.0031182

**Published:** 2012-02-10

**Authors:** Sven Vanneste, Kathleen Joos, Dirk De Ridder

**Affiliations:** Brai^2^n, TRI & Department of Neurosurgery, University Hospital Antwerp, Belgium; University of Bern, Switzerland

## Abstract

**Background:**

Tinnitus refers to auditory phantom sensation. It is estimated that for 2% of the population this auditory phantom percept severely affects the quality of life, due to tinnitus related distress. Although the overall distress levels do not differ between sexes in tinnitus, females are more influenced by distress than males. Typically, pain, sleep, and depression are perceived as significantly more severe by female tinnitus patients. Studies on gender differences in emotional regulation indicate that females with high depressive symptoms show greater attention to emotion, and use less anti-rumination emotional repair strategies than males.

**Methodology:**

The objective of this study was to verify whether the activity and connectivity of the resting brain is different for male and female tinnitus patients using resting-state EEG.

**Conclusions:**

Females had a higher mean score than male tinnitus patients on the BDI–II. Female tinnitus patients differ from male tinnitus patients in the orbitofrontal cortex (OFC) extending to the frontopolar cortex in beta1 and beta2. The OFC is important for emotional processing of sounds. Increased functional alpha connectivity is found between the OFC, insula, subgenual anterior cingulate (sgACC), parahippocampal (PHC) areas and the auditory cortex in females. Our data suggest increased functional connectivity that binds tinnitus-related auditory cortex activity to auditory emotion-related areas via the PHC-sgACC connections resulting in a more depressive state even though the tinnitus intensity and tinnitus-related distress are not different from men. Comparing male tinnitus patients to a control group of males significant differences could be found for beta3 in the posterior cingulate cortex (PCC). The PCC might be related to cognitive and memory-related aspects of the tinnitus percept. Our results propose that sex influences in tinnitus research cannot be ignored and should be taken into account in functional imaging studies related to tinnitus.

## Introduction

Subjective tinnitus is a condition in which a patient perceives an auditory phantom sound that can take the form of ringing, buzzing, roaring or hissing in the absence of an external sound [1]. This is also referred to as a phantom auditory sensation. In 5 to 15% of the population, this tinnitus sensation is unremitting and it is estimated that for 2–3 in 100 this auditory phantom percept severely affects the quality of life as tinnitus causes a considerable amount of distress [2].

Studying the affective dimension of tinnitus, distress has been considered as an aversive state in which a person is unable to adapt completely to stressors (i.e. tinnitus) and shows maladaptive behaviors [3], [4]. It has been suggested that females are more influenced by distress in comparison to males, as different assessment instruments capture females and male reactions to the stressor in a different way. Typically, pain, sleep, and energy, are perceived to be significantly more severe by female tinnitus patients [5]. However, these symptoms can also have an influence on tinnitus perception. As such, tinnitus distress and other severe problems might influence each other in both directions. In addition, women tend to show more hypersensitivity to aversive musical stimuli [6]. Females also tend to respond to distress more with rumination, a coping method that focuses on internal feelings rather than by externally oriented direct actions for stress reliefs [7]. Further studies on gender differences in emotional regulation indicate that females with high depressive symptoms show greater attention to emotion, with less anti-rumination emotional repair strategies than males [8].

Several neuroimaging studies have reported gender differences in brain locations involved both in perception [9], subjective experience of emotion [10] and emotion regulation [11]. Particularly, neural activity in the orbitofrontal cortex, the anterior cingulate cortex, the insula and the amygdala [11], [12] have shown to differ between sexes during emotional processing and emotional regulation. These brain areas are also important in tinnitus perception and tinnitus-related distress [13]–[16]. Furthermore, functional differences in the neural mechanism between sexes in auditory processing have been demonstrated [17]. For example, differences in the primary auditory cortex activation exist between males and females in silent lip reading [18] as well as in processing of noise stimuli [19]. In addition, women demonstrate a greater overall structural connectivity of the underlying organization of their cortical networks than men [20]. Sex-related functional connectivity differences were found in the amygdala as well during an eyes closed, “resting” condition [21].

Given the different prevalence's of affective disturbances based on tinnitus perception and tinnitus-related distress, we hypothesized that male and female tinnitus patients might show different patterns of neural activity and connectivity. We therefore focus on the differences in cortical sources of the resting-state EEG (eyes closed) between male and female tinnitus patients using continuous scalp EEG recordings and Low Resolution Electromagnetic Tomography (sLORETA), a tomographic inverse solution imaging technique [22].

## Results

### Tinnitus Questionnaire and Beck Depression Inventory

A comparison between males (*M* = 54.51, *Sd* = 14.91) and females (52.60, *Sd* = 15.43) with tinnitus on the TQ showed no significant effect (*F*(1,34) = .16, *p* = .70), while the BDI–II revealed that females had a higher mean score (*M* = 14.56, *Sd* = 13.20) than males (*M* = 7.83, *Sd* = 5.40) (*F*(1,34) = 4.10, *p*<.05). In addition, we further explore whether males and females differ on the different subscales of the TQ, namely emotional and cognitive distress, intrusiveness, auditory perceptual difficulties, sleep disturbances and somatic complaints. However, no significant gender differences were obtained (*p*>.40).

### Source localization

**Figure 1:**
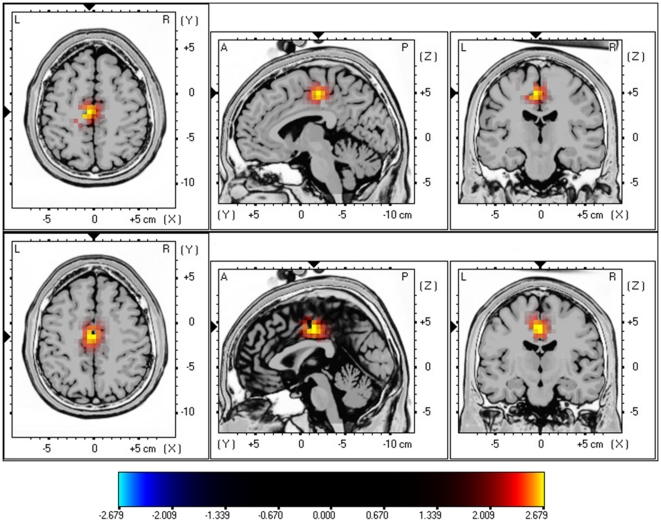
sLoreta constrast analysis between tinnitus versus control (*p*<.05). Increased neural synchonization within Beta1 (13–18 Hz; Top Panel) and Beta2 (18.5–21 Hz; Bottom Panel) in the posterior cingulate cortex (BA23).

A comparison was made between tinnitus patients and a control group. This analysis revealed a significant difference for the beta1 and beta2 frequency band demonstrating that tinnitus patients have an increased activity in the posterior cingulate cortex (see Figure 1; *P*<.05). No significant differences could be retrieved in the delta, theta, alpha1, alpha2, beta3 and gamma frequency bands.

A similar analysis comparing females (control+tinnitus patients) and males (control+tinnitus patients) did not obtain significant effects for delta, theta, alpha1, alpha2, beta1, beta2, beta3 and gamma frequency bands.

**Figure 2:**
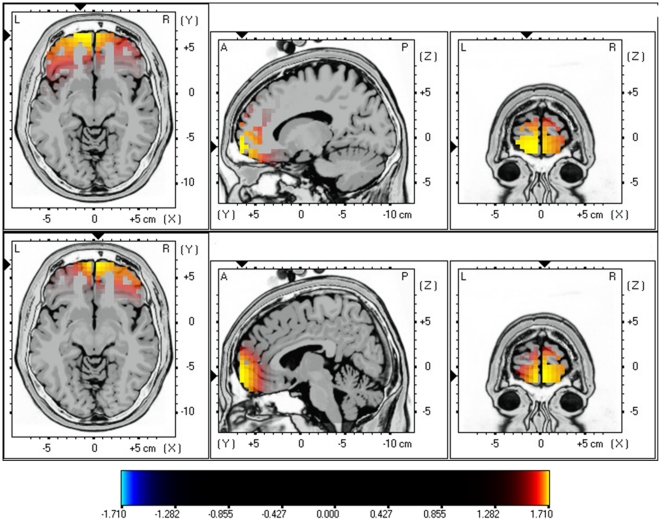
sLORETA contrast analysis between female versus male tinnitus patients (*p*<.05). Increased neural synchronization within Beta1 (13–18 Hz; Top Panel) and Beta2 (18.5–21 Hz; Bottom Panel) in the orbitofrontal cortex (OFC; BA10 and BA11).

sLORETA showed significant differences between female and male tinnitus patients. Increased synchronized beta1 and beta2 activity could be revealed in the orbitofrontal (BA11) extending to frontopolar (BA10) cortex for females in comparison to males (see Figure 2; *P*<.05). No significant differences could be retrieved in the delta, theta, alpha1, alpha2, beta3 and gamma frequency bands.

A comparison between males and females control subjects did not obtain significant effects for delta, theta, alpha1, alpha2, beta1, beta2, beta3 and gamma frequency bands.

**Figure 3:**
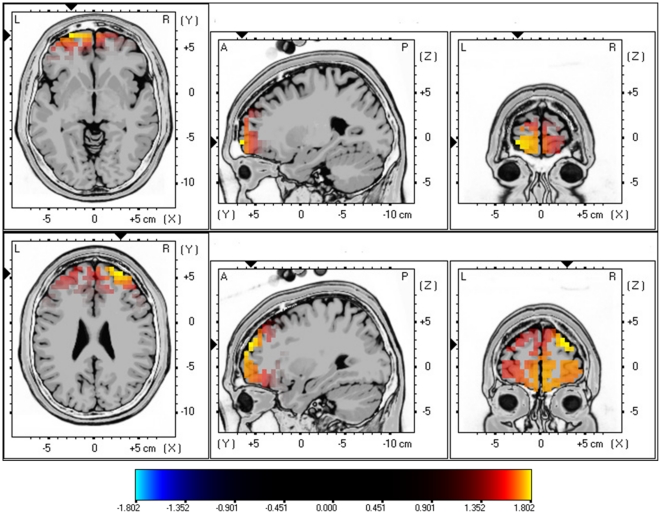
sLORETA contrast analysis between Females with tinnitus versus Female controls (*p*<.05). Increased neural synchronization within Beta1 (13–18 Hz; Top Panel) and Beta2 (18.5–21 Hz; Bottom Panel) in the orbitofrontal cortex (OFC; BA10 and BA11).

Next the tinnitus patients are compared with gender and age-matched control group for beta1 and beta2 activity. Analysis revealed again increased synchronized beta1 and beta2 activity in the frontopolar and orbitofrontal cortex (BA10 & BA11) for females with tinnitus in comparison to female controls (see Figure 3; *P*<.05). No significant differences could be retrieved in the delta, theta, alpha1, alpha2, beta1, beta3 and gamma frequency bands.

**Figure 4:**
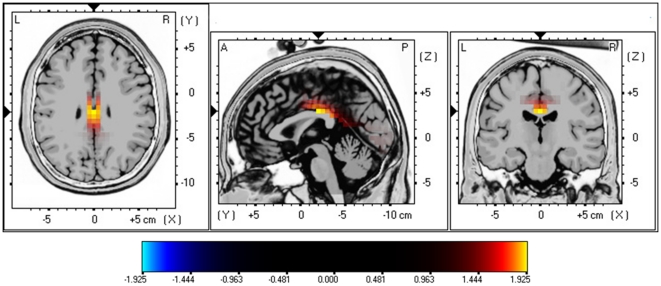
sLoreta constrast analysis between tinnitus versus control males (*p*<.05). Increased neural synchonization within Beta3 (21.5–30 Hz; Top Panel) in the posterior cingulate cortex (BA23).

A similar analysis for males with tinnitus in comparison to male controls did obtain a significant effect for beta3 (see Figure 4; *P*<.05) demonstrating increased activity in the posterior cingulate cortex (PCC; BA23) for male tinnitus patients. No significant differences could be retrieved in the delta, theta, alpha1, alpha2, beta1, beta2 and gamma frequency bands.

### Correlations

**Figure 5:**
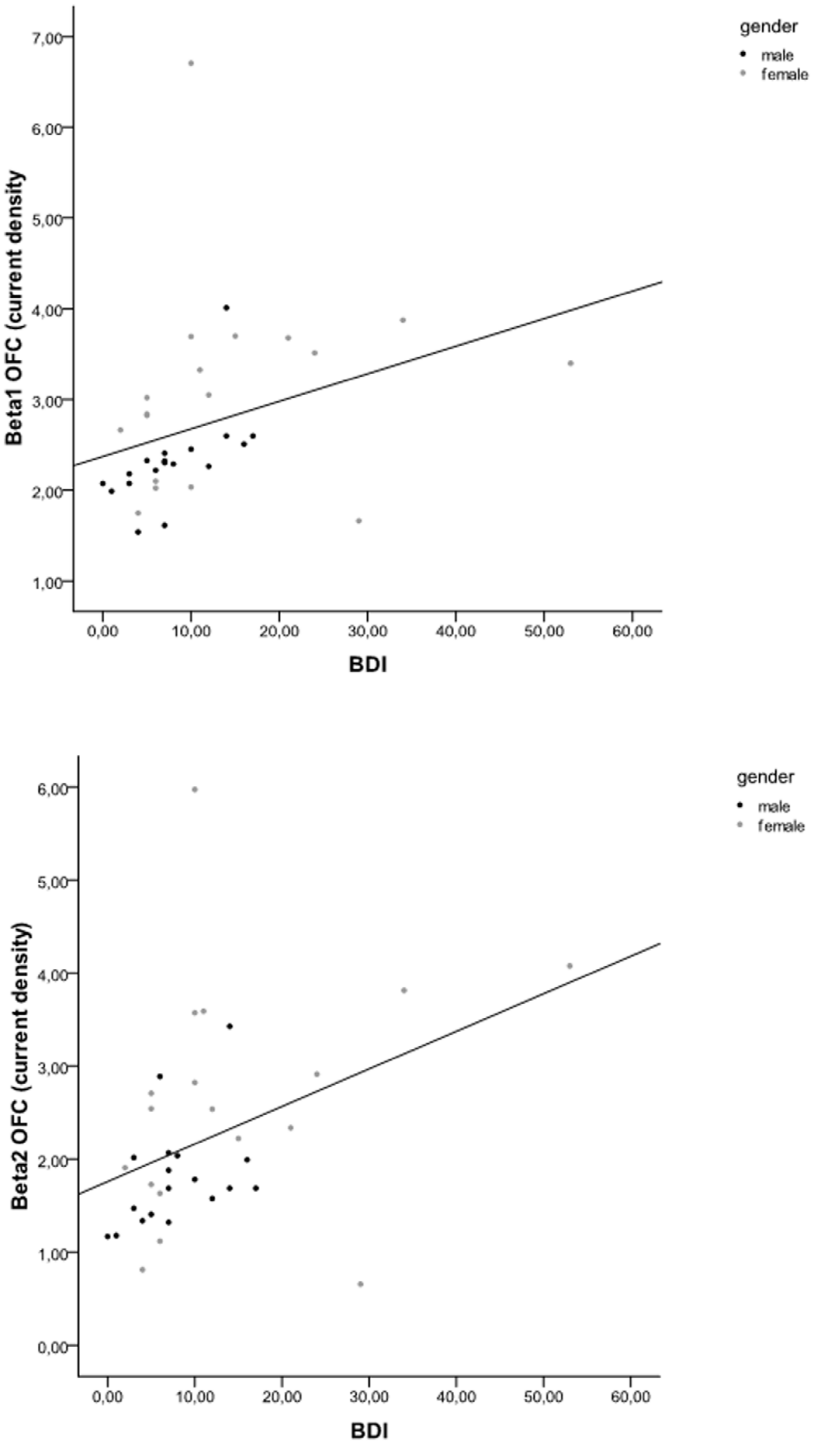
Pearson correlation between BDI and respectively Beta1 Orbifrontal cortex and Beta2 Orbitofrontal cortex.

Pearson correlation revealed that the BDI correlates with the Orbitofrontal cortex beta1 (r = .34, *p*<.05) and beta2 (r = .39, *p*<.05), respectively (see Figure 5). In addition, a similar analysis was conducted excluding outliers, i.e. based on visual inspection one could argue that 3 females score higher on the BDI in comparison to the other males and females. However, after excluding these possible outliers still a positive correlation was obtained between the BDI correlates with the orbitofrontal cortex beta1 (r = .46, *p*<.05) and beta2 (r = .35, *p*<.05).

### Region of interest analysis

#### 1. Orbitofrontal cortex

**Figure 6:**
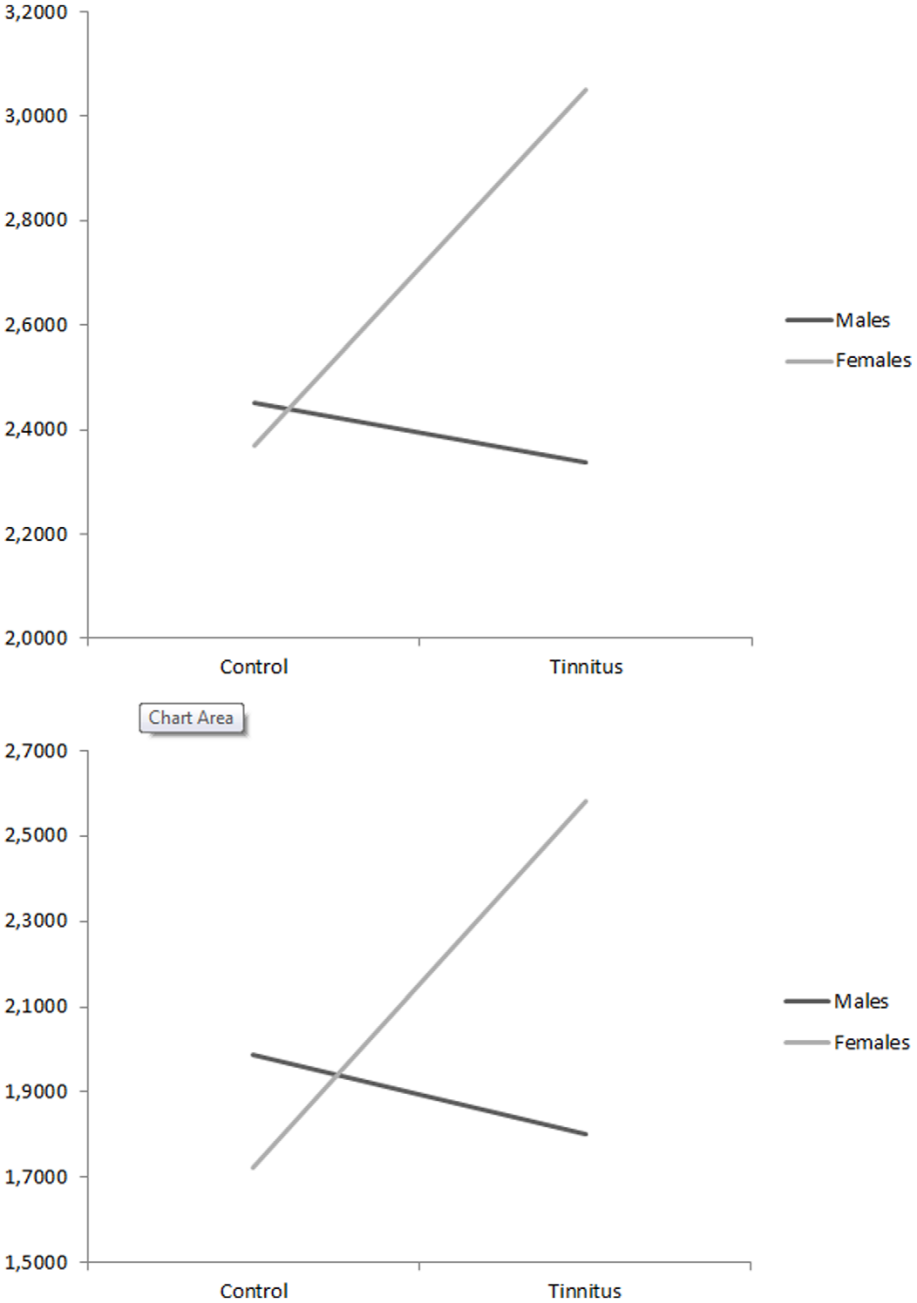
Region of interest analysis for the Orbitofrontal cortex for respectively beta1 and beta2 frequency band (log-transformed current density).

A two-way MANOVA with gender (males vs. females)×group (tinnitus vs. control) as independent variables and log-transformed current density for the different groups on the orbitofrontal cortex (left and right) for the beta1 and beta2 frequency band was conducted (see Figure 6). Analysis revealed no significant main effect for respectively gender *F*(2,67) = 1.39, *p* = .26 and group *F*(2,67) = 1.37, *p* = .26. However an interaction effect was obtained for gender (males vs. females)×group (tinnitus vs. control), *F*(2,67) = 3.14, *p*<.05. An univariate ANOVA further showed that for both beta1 (*F*(1,68) = 4.48, *p*<.05) and beta2 (*F*(1,68) = 5.78, *p*<.05) frequency bands a significant interaction effect was obtained. For both beta1 (*F*(1,68) = 7.20, *p*<.01) and beta2 (*F*(1,68) = 6.43, *p*<.05) contrast analysis revealed that females with tinnitus have an increased log-transformed current density in comparison to female control subjects. In addition, a contrast analysis for beta1 (*F*(1,68) = 6.75, *p*<.05) and beta2 (*F*(1,68) = 8.08, *p*<.01) demonstrated increased log-transformed current density for female tinnitus patients in comparison to male tinnitus patient. For both beta1 and beta2 no significant effects were obtained when comparing males with tinnitus and male control subjects as well as a comparison between female and male control subjects.

#### 2. Auditory cortex

**Figure 7:**
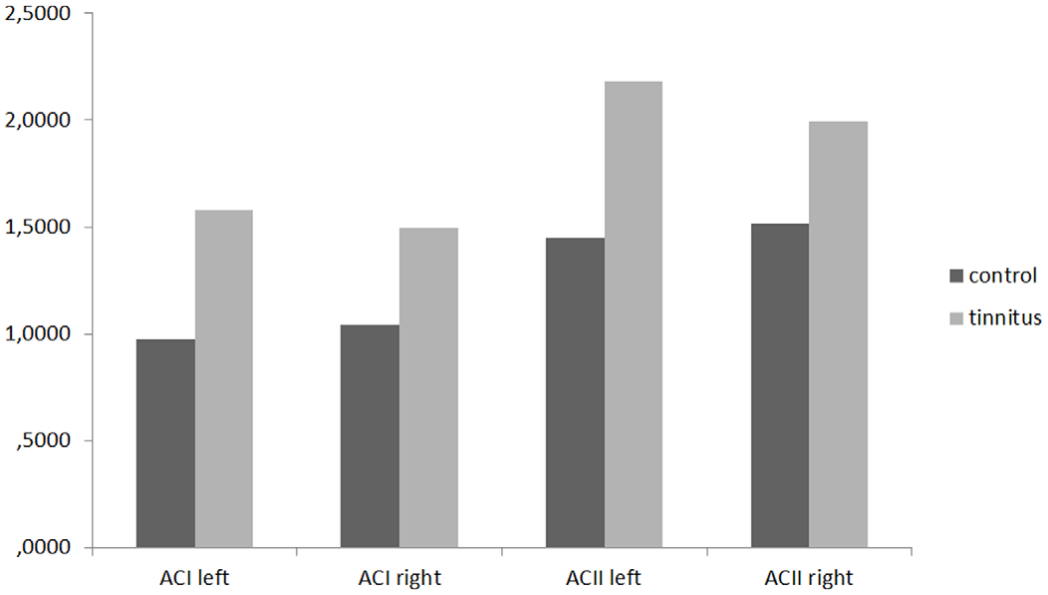
Region of interest analysis for the left and right primary and secondary auditory cortex for gamma band frequency (log-transformed current density).

A significant effect was found for the log-transformed current density for the different groups on the region of interest (auditory cortex) for the gamma frequency band, *F*(4,65) = 5.38, *p*<.001 (see Figure 7). Univariate ANOVA further yielded a significant effect for respectively left primary auditory cortex (*F*(1, 68) = 19.34, *p*<.001), right primary auditory cortex (*F*(1,68) = 11.37, *p*<.001), left secondary auditory cortex (*F*(1, 68) = 17.83, *p*<.001), right secondary auditory cortex (*F*(1, 68) = 5.79, *p*<.05) indicating that the control subjects had significant lower log averaged current density in comparison to tinnitus patients. No significant effect was obtained for the comparison of females and males (*F*(4,65) = 1.55, *p* = .22) and no interaction effect (gender (males vs. females)×group (tinnitus vs. control)) (*F*(4,65) = 1.08, *p* = .37) was obtained as well. No significant differences were found for delta, theta, alpha2, beta1, beta2, and beta3 bands.

### Hierarchical regression analyses

In order to investigate the relative importance of both gender and BDI on the OFC for respectively beta1 and beta2, hierarchical regression analyses with OFC for respectively beta1 and beta2 as the dependent variable and BDI and gender as independent variables were conducted. First we compared BDI and gender by alternatively including the variables in the first and second block. A first analysis revealed a significant effect for BDI on OFC Beta1 (*F* = 4.12, *p*<.05, R^2^ = .11). After including gender to the modal a significant effect was obtained (*F* = 4.44, *p*<.05, R^2^ = .21) revealing that gender significantly contributed to the model (*F_change_* = 4.34, *p*<.05, ΔR^2^ = 10%). A similar analysis for the OFC in Beta2 revealed a significant effect for BDI on OFC Beta2 (*F* = 6.24, *p*<.05, R^2^ = .15). After including gender to the modal a significant effect was obtained (*F* = 4.49, *p*<.05, R^2^ = .23) demonstrating that gender significantly, however marginal, contributed to the model (*F_change_* = 2.96, *p*<.10, ΔR^2^ = 8%).

### Functional connectivity

**Figure 8:**
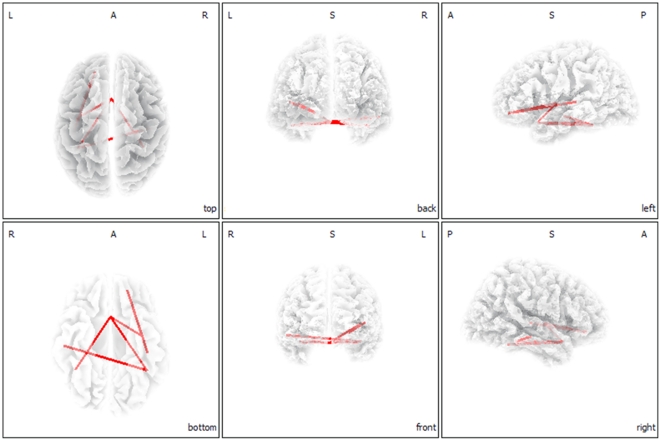
Connectivity contrast analysis between female and males tinnitus patients (*p*<.05). Increased neural synchronization within alpha 1 (8–10 Hz) connectivity for female tinnitus patients.

Functional connectivity analysis yielded a significant difference between female and male tinnitus patients in the alpha1 frequency band (*P*<.05). Females demonstrated increased functional connectivity in comparison to males between both left and right parahippocampus and subgenual anterior cingulate cortex (sgACC), and from the sgACC connectivity to the left insula (see Figure 8). From the left insula also increased functional connectivity was found to the left OFC. Furthermore, a functional connection for alpha1 was shown between the left OFC and left secondary auditory cortex (lA2) as well as between the left parahippocampal area and the right primary auditory cortex (rA1). No significant differences were found for delta, theta, alpha2, beta1, beta2, beta3 and gamma bands.

Functional connectivity analysis between female and male control patients yielded no significant effects for respectively delta, theta, alpha1, alpha2, beta1, beta2, beta3 and gamma bands.

### Brain topography and Power Spectra

**Figure 9:**
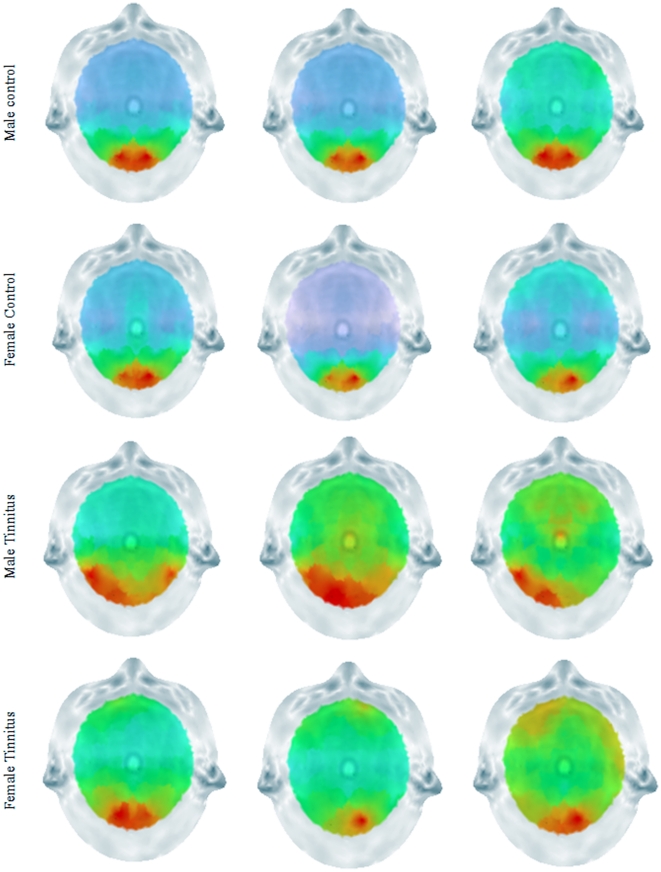
Scalp topography for Beta1, Beta2 and Beta3 for respectively Male Controls, Female Controls, Males Tinnitus patients and Female Tinnitus Patients.

**Figure 10:**
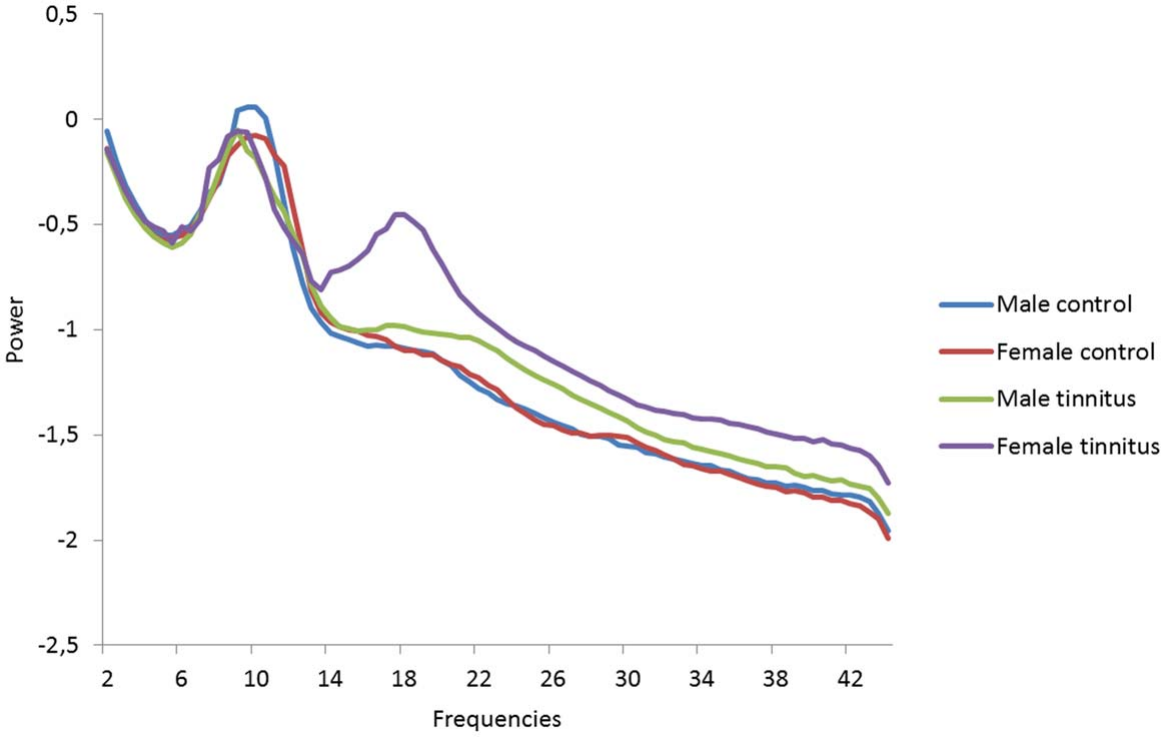
Power spectrum analysis for respectively Male Controls, Female Controls, Males Tinnitus patients and Female Tinnitus patients.

To verify whether the obtained results in the OFC are not artifact related both brain topographies and a power spectrum analysis was conducted. The topographies demonstrate that most power was retrieved occipitally and not frontally for male and female control subjects as well as male and female tinnitus patients (see Figure 9). The distribution for the frontal electrodes was similar across the different groups over the different frequency bands. For the beta band female tinnitus patients have an increased power from 14 to 22 Hz. However, these differences were not significant in comparison to the other group (see Figure 10).

## Discussion

This study investigated gender-specific neural correlates of tinnitus. It was demonstrated that tinnitus patients had an increased activity for beta1 and beta2 in the PCC compared to a control group. In addition, the tinnitus revealed differences in comparison to the control group for gamma activity in primary and secondary auditory cortex. Female tinnitus patients further demonstrated differences from male tinnitus patients in the OFC (BA11) extending to the frontopolar (BA10) for beta1 and beta2 oscillatory activity. These results confirm a recent bifrontal tDCS study where females showed better responses than males on their tinnitus [23].

In addition, when comparing to a control group of females, female tinnitus patients showed more frontopolar OFC for beta1 and beta2 oscillatory activity, These findings were further confirmed by a interaction effect indicating that specifically female tinnitus patients had an increased beta1 and beta2 activity in the frontopolar and OFC in comparison to male tinnitus patients, as well as to a male and female control group. Also a correlation was obtained between activity in beta1 and beta2 frequency bands in the OFC and mood measured with the BDI. But even when controlling for mood, the OFC beta activity correlated with gender, suggesting that the OFC beta activity does differentiate female from male patients. Comparing male tinnitus patients to a control group of males significant differences could be found for beta3 oscillatory activity in the PCC. In addition, we established differences in functional connectivity between the different regions of interest for alpha1 band. Female tinnitus patients yielded increased connectivity between left and right parahippocampus and sgACC, and from the sgACC connectivity to the left insula (see Fig. 3). From the left insula also increased connectivity was found to the left OFC. Furthermore, a functional connection for alpha1 was shown between the left OFC and left secondary auditory cortex (lA2) as well as between the left parahippocampal area and the right primary auditory cortex (rA1).

A main concern might be that the difference found in the frontopolar and OFC might be artefact related. However, our results firstly demonstrated that there was a clear correlation between OFC and BDI. In addition, brain topography analyses showed that most power was located occipital for all groups. If frontopolar and OFC differences were artefact related one would expect more activity frontally, as muscle related activity is usually broader and generates more power [24]. A specific analysis on the frontal electrodes (FP1, FP2, F7, F8) showed an increased power from female tinnitus patients within the 14–22 Hz window, however these results were not significant. The power spectra show a typical distribution for the different groups on the specific electrodes indicating that the activity measured might be not artefact related.

This study demonstrated that female tinnitus patients had a higher score on the BDI–II than male tinnitus patients for the same amount of tinnitus related distress and the same tinnitus intensity. Differences on BDI–II between males and females have already been described in previous literature, showing that women in general have significant higher depression severity scores than men [25], [26]. In this study the BDI scores are low in comparison to the depression literature [25], [26], but similar to other tinnitus studies [27].

The posterior cingulate cortex might be related to cognitive and memory related aspects of the tinnitus percept, as the PCC is involved in cognitive aspects of auditory processing [28]. Activity in the posterior cingulate cortex has indeed been linked to successful retrieval from auditory (and visual) memory [29], [30]. The PCC has been proposed to exert a salience based cognitive auditory comparator function [28]. When the PCC component is deficient or less active, such as in tinnitus distress [31], this could reflect an incapacity of the PCC/precuneus to exert its salience based comparator function, pulling irrelevant auditory (tinnitus) sound from hippocampal memory [32], via dysfunctional parahippocampal auditory sensory gating [33], analogous to what has been proposed for auditory hallucinations [34].

Previous research has already shown that the OFC is important for emotional processing of sounds [35]–[38]. For example, patients with OFC lesions had reduced self-evaluated perception of the unpleasantness of the acoustic probe stimulus [39]. Koch et al. also found that an interaction between negative emotion and working memory in females involved activation in the OFC, suggesting that during the control of emotion, females mainly recruit the emotion-associated areas [40]. Furthermore, the OFC is known to serve as a store of implicitly acquired linkages between factual knowledge and bio-regulatory states, including those that constitute feelings and emotions [41]. Female tinnitus patients have been found to be more emotionally responsive to tinnitus-related distress [42] as well as in physiological responses to negative emotional stimuli than males [43]. We found similar results, as both males and females had a similar score on the tinnitus questionnaire which measure tinnitus related distress, but scored differently on the BDI.

The OFC together with the insula plays a key role in the top-down modulation of automatic or peripheral physiological responses to emotional experiences [44], [45]. The increased synchronized alpha connectivity between the OFC and the insula for females might represent a modulation of the autonomic physiological responses evoked by tinnitus. The OFC connects with other limbic areas important for processing of emotion [46].

This study also finds an indirect increased functional connectivity between the OFC and the sgACC. The sgACC has previously been associated with negative affect [47], processing of aversive sounds [48] and unpleasant music [38] as well as tinnitus [16] and tinnitus related distress. As such, tinnitus and tinnitus-related distress might have stronger impact on OFC in females.

An increased functional connectivity between the parahippocampal areas and the sgACC for females is demonstrated in our study. The parahippocampal area has strong functional connections with the sgACC [31], [49] which changes under distress [50]. It also has strong functional connections with the amygdala [49], involved in emotional auditory memory [51], [52]. The role of the parahippocampal areas in the network might be related to a constant updating of the tinnitus sound from emotional memory, preventing habituation [32]. The hippocampal involvement in auditory habituation is already demonstrated in auditory sensory gating studies [33]. Gender differences in auditory sensory gating have been described: females had less inhibitory mechanisms acting on the generator substrates [53], which might also be present in our study.

Tinnitus is characterized by an ongoing abnormal spontaneous activity [54] and reorganization [55] of the auditory cortex. The auditory cortex encodes tinnitus intensity [56]. Sex differences were already detected in processing noise, indicating that females activate their auditory cortex significantly more than males [19]. However, this study did not reveal any activity differences in the auditory cortex. This fits with the fact that tinnitus intensity was not significantly different between females and males. But females demonstrated increased functional connectivity between the auditory cortex and the OFC. As the auditory cortex is important in tinnitus and more specifically in the tinnitus intensity [56], a direct functional connection with the OFC might further enforce emotional reactivity to the phantom sound.

Gender differences were found in the OFC that might explain why in females the same tinnitus related distress and the same tinnitus intensity induces more depressive feelings than in males, as was found on the BDI. Yet, since depression is more frequent in women, the higher depression scores in the female tinnitus group could be completely unrelated to the presence of tinnitus.

Additional research is needed to further explore the differences between male and female tinnitus patients. The strength of this study is that we conducted our analyses on a very homogeneous group of tinnitus patients, namely patients with narrow band noise bilateral tinnitus. However, previous research already showed that there are cortical differences between unilateral and bilateral tinnitus [57], as well as pure tone and narrow band noise tinnitus [58]. Based on these findings additional research is needed to further explore gender effects in tinnitus type and mood to generalize the results.

In summary, the increased beta activity in the prefrontal cortex (BA11-10) with its increased functional alpha connectivity between the OFC, insula, sACC, parahippocampal areas and the auditory cortex in females explains that tinnitus related auditory cortex activity is functionally connected to auditory emotion related areas resulting in more a depressive state even though the tinnitus intensity and tinnitus related distress are not different from men. Male tinnitus patients have more beta activity in the PCC which might be related to cognitive and memory related aspects of the tinnitus percept. As such, our results clearly show that sex influences in tinnitus research cannot be ignored and should be taken into account in functional imaging studies related to tinnitus.

## Methods

### Participants

Thirty-six patients with narrow band noise bilateral tinnitus (N = 36; 18 males and 18 females) with a mean age of 51.47 (SD = 12.95 years) were selected from the multidisciplinary Tinnitus Research Initiative (TRI) Clinic of the University Hospital of Antwerp, Belgium. Individuals with pulsatile tinnitus, Ménière disease, otosclerosis, chronic headache, neurological disorders such as brain tumors, and individuals being treated for mental disorders were not included in the study in order to obtain a homogeneous sample. No significant differences were found between males and females for tinnitus duration, VAS intensity, and tinnitus related distress based upon the tinnitus questionnaire (see Table 1).

**Table 1:** Patients' characteristics.

	Gender		
	Females	Males	*p-values*
Age	50.82	52.12	.70
Tinnitus duration	4.96	5.23	.72
VAS intensity	6.54	6.87	.53
Hearing Loss (HL)	26.94	23.98	.68
Tinnitus pitch	26.94	23.89	.44

All patients were investigated for the extent of hearing loss using audiograms. Tinnitus matching was performed looking for tinnitus pitch (frequency) and tinnitus intensity. No significant differences were found for hearing loss between males and females, as measured by the loss in decibels (dB HL) at the tinnitus frequency.

Participants were requested to refrain from alcohol consumption 24 hours prior to recording and from caffeinated beverages on the day of recording.

Subjective depression was assessed with the Beck Depression Inventory-revised (BDI–II), a 21-item self-report instrument with good psychometric properties [59].

This study was approved by the local ethical committee (Antwerp University Hospital) and was in accordance with the declaration of Helsinki. Patients gave oral informed consent before the procedure. The EEG was obtained as a standard procedure for diagnostic and neuromodulation treatment purposes.

### EEG recording

EEG recordings were obtained in a fully lighted room with each participant sitting upright on comfortable chair. The actual recording lasted approximately 5 min. The EEG was sampled with 19 electrodes (Fp1, Fp2, F7, F3, Fz, F4, F8, T7, C3, Cz, C4, T8, P7, P3, Pz, P4, P8, O1 O2) in the standard 10–20 International placement referenced to linked ears and impedances were checked to remain below 5 kΩ. Data were collected eyes-closed (sampling rate = 1024 Hz, band passed 0.15–200 Hz). Data were resampled to 128 Hz, band-pass filtered (fast Fourier transform filter) to 2–44 Hz and subsequently transposed into Eureka! Software [60], plotted and carefully inspected for manual artifact-rejection. All episodic artifacts including eye blinks, eye movements, teeth clenching, body movement, or ECG artifact were removed from the stream of the EEG. In addition, an independent component analysis (ICA) was conducted to further verify if all artifacts were excluded. To investigate the effect of possible ICA component rejection we compared the power spectra in two approaches: (1) after visual artifact rejection only (before ICA) and (2) after additional ICA component rejection (after ICA). To test for significant differences between the two approaches we performed a repeated-measure ANOVA, considering mean band power as within-subject variable and groups (unilateral vs. bilateral tinnitus) as between-subject variable. The mean power in delta (2–3.5 Hz), theta (4–7.5 Hz), alpha1 (8–10 Hz), alpha2 (10–12 Hz), beta1 (13–18 Hz), beta2 (18.5–21 Hz), beta3 (21.5–30 Hz) and gamma (30.5–45 Hz) did not show a statistically significant difference between the two approaches. Therefore, we continue by reporting the results of ICA corrected data. Average Fourier cross-spectral matrices were computed for bands delta (2–3.5 Hz), theta (4–7.5 Hz), alpha1 (8–10 Hz), alpha2 (10–12 Hz), beta1 (13–18 Hz), beta2 (18.5–21 Hz), beta3 (21.5–30 Hz) and gamma (30.5–44 Hz).

### Control database subjects

Similarly to the tinnitus patients, EEGs (Mitsar, Nova Tech EEG, Inc, Mesa) were selected from an in-house normative database as a control group (N = 36; 18 males and 18 females) with a mean age of 51.47 (SD = 12.95 years). Recordings were made in similar circumstances, i.e.e in a fully lighted room with each participant sitting upright in a comfortable chair. None of these subjects were known to suffer from tinnitus or hearing loss. Exclusion criteria for the control subjects were known psychiatric or neurological illness, drug/alcohol abuse, current psychotropic/CNS active medications, history of head injury (with loss of consciousness) or seizures. The EEG was sampled with 19 electrodes (Fp1, Fp2, F7, F3, Fz, F4, F8, T7, C3, Cz, C4, T8, P7, P3, Pz, P4, P8, O1 O2) in the standard 10–20 International placement referenced to linked lobes and impedances were checked to remain below 5 kΩ. Data were collected for 100 2-s epochs eyes closed, sampling rate = 1024 Hz, and band passed from 0.15 to 200 Hz. Data were resampled to 128 Hz, band-pass filtered (fast Fourier transform filter) to 2–44 Hz. The data were cleaned-up in a similar way to the tinnitus patients by manual artifact rejection and ICA. Again to investigate the effect of possible ICA component rejection we compared the power spectra in two approaches: (1) after visual artifact rejection only (before ICA) and (2) after additional ICA component rejection (after ICA). To test for significant differences between the two approaches we performed a repeated-measure ANOVA, considering mean band power as within-subject variable.

### Source localization

Standardized low-resolution brain electromagnetic tomography (sLORETA) was used to estimate the intracerebral electrical sources that generated the scalp-recorded activity in each of the eight frequency bands [61]. sLORETA computes electric neuronal activity as current density (A/m^2^) without assuming a predefined number of active sources. The sLORETA solution space consists of 6,239 voxels (voxel size: 5×5×5 mm) and is restricted to cortical gray matter and hippocampi, as defined by digitized MNI152 template [62]. Scalp electrode coordinates on the MNI brain are derived from the international 5% system [63]. The tomography sLORETA has received considerable validation from studies combining LORETA with other more established localization methods, such as functional Magnetic Resonance Imaging (fMRI) [64], [65], structural MRI [66], Positron Emission Tomography (PET) [67]–[69]. Further sLORETA validation has been based on accepting as ground truth the localization findings obtained from invasive, implanted depth electrodes, in which case there are several studies in epilepsy [70], [71] and cognitive ERPs [72]. It is worth emphasizing that deep structures such as the anterior cingulate cortex [73], and mesial temporal lobes [74] can be correctly localized with this method.

### Functional connectivity

Coherence and phase synchronization between time series corresponding to different spatial locations are usually interpreted as indicators of the “functional connectivity”. However, any measure of dependence is highly contaminated with an instantaneous, non-physiological contribution due to volume conduction and low spatial resolution (Pascual-Marqui, 2007a). Therefore Pascual-Marqui, (2007b) introduced a new technique (i.e. Hermitian covariance matrices) that removes this confounding factor considerably. As such, this measure of dependence can be applied to any number of brain areas jointly, i.e. distributed cortical networks, whose activity can be estimated with sLORETA. Measures of linear dependence (coherence) between the multivariate time series are defined. The measures are expressed as the sum of lagged dependence and instantaneous dependence. The measures are non-negative, and take the value zero only when there is independence of the pertinent type and are defined in the frequency domain: delta (1–3.5 Hz), theta (4–7.5 Hz), alpha1 (8–10 Hz), alpha2 (10–12 Hz), beta1 (13–18 Hz), beta2 (18.5–21 Hz), beta3 (21.5–30 Hz) and gamma (30.5–45 Hz). Based on this principle lagged linear connectivity was calculated. Regions of interest were defined based on previous brain research on tinnitus (see table 2 for overview) and the present findings based on the source analysis (e.g. orbitofrontal cortex and posterior cingulate cortex).

**Table 2:** Regions of Interest.

		Authors
Anterior Cingulate Cortex	Dorsal	[14] [13]
	Subgenual	[16]
Auditory Cortex		[55] [76] [15] [54]
Dorsal Lateral Prefrontal Cortex		[77]
Insula		[15] [31]
Parahippocampus		[78]

### Statistical analysis

In order to identify potential differences in brain electrical activity between groups, sLORETA was then used to perform voxel-by-voxel between-condition comparisons of the current density distribution. Nonparametric statistical analyses of functional sLORETA images (statistical non-parametric mapping; SnPM) were performed for each contrast employing a t-statistic for unpaired groups and a corrected (P<0.05). As explained by Nichols and Holmes, the SnPM methodology does not require any assumption of Gaussianity and corrects for all multiple comparisons [75]. We performed one voxel-by-voxel test (comprising 6,239 voxels each) for the different frequency bands.

### Region of interest analysis

The log-transformed electric current density was averaged across all voxels belonging to the region of interest, respectively left and right primary auditory cortex (BA40 and BA41) and left and right secondary auditory cortex(BA21 and BA22) separately for the gamma frequency band. Also the region of interest analysis was performed for the frontopolar and orbitofrontal cortex (BA10 & BA11).

A multivariate ANOVA (i.e. Wilks' Lambda) for the frequency bands was used with the respective region of interest (i.e. left and right primary auditory cortex (BA40 and BA41) and left and right secondary auditory cortex (BA21 and BA22) as dependent variables and different groups (pure tone, narrow band noise and control subjects) as independent variable. A Bonferroni correction was applied for multiple comparisons.

A Pearson correlation was calculated between BDI and frontopolar and orbitofrontal cortex (BA10 & BA11). Also a hierarchical regression analysis was conducted with BDI and gender as independent variables and frontopolar and orbitofrontal cortex (BA10 & BA11) as dependent variable. The aim was to verify whether gender significantly contributed to the model.

### Brain topography and Power Spectra

A digital FFT-based power spectrum analysis (Time Domain Tapering: Hamming, Frequency Domain Smoothing: Blackman, Overlapping FFT Windows Advancement Factor: 8) computed the power density of EEG rhythms with 0.5 Hz frequency resolution.

In order to verify whether the findings obtained are not related to artifacts (i.e. eye movements and muscle activity) we verified the power spectral analysis for the 4 frontal electrodes (FP1, FP2, F7, F8). To summarize the data and because spectra from all electrodes demonstrated similar shape and scale, we averaged the log transformed spectra of all 4 scalp electrodes for each subject. We then averaged these individual spectra to one spectrum for respectively male controls, female controls, male tinnitus patients and female tinnitus patients. In addition also the brain topographies for beta 1, beta2, and beta3 were calculated to further detect possible artifacts.
